# Advances in preparation of acellular human dermis for tissue banking and transplantation

**DOI:** 10.1007/s10561-024-10153-0

**Published:** 2024-12-10

**Authors:** Irit Stern, Valentina Barrera, Michael Randles, Paul Rooney

**Affiliations:** 1 NHS Blood and Transplant, Tissue Services, 14 Estuary Banks, Speke, Liverpool, L24 8RB UK; 2 NHS Blood and Transplant, Tissue Services R&D, 14 Estuary Banks, Speke, Liverpool, L24 8RB UK; 3 Faculty of Medicine and Life Sciences, Chester Medical School, University of Chester, Chester, UK

**Keywords:** Skin, Decellularised, Dermis, Benzonase, Pulmozyme, Allograft

## Abstract

Non-healing wounds cost the National Health Service over £5.6 billion annually in wound management. Skin allografts are used to treat non-healing wounds, ulcers and burns, offering the best protection against infection. In order to allow host cells to repopulate and to avoid immunogenicity, cell components are removed through decellularisation. Decellularisation of human dermis has so far been performed in NHS Blood and Transplant using a combination of two enzymes (RNase T1 and the recombinant human DNase Pulmozyme)®. This study aims at validating a new method to remove DNA from donated dermis via the use of a single enzyme, Benzonase, known for its effectiveness of DNA digestion. Skin samples were decellularised by removing the epidermis, lysing of dermal cells, removal of cellular fragments by a detergent wash and removal of nucleic acids by a nuclease incubation with either Benzonase or Pulmozyme + RNase T1. DNA quantification with PicoGreen, as well as histology on wax-embedded biopsies, stained with DAPI and haemotoxylin and eosin, were performed. In vitro toxicity test on human osteosarcoma immortalised cells and skin fibroblasts, and biomechanical (tensile) testing, were also performed. The effectiveness of DNA digestion with the new methodology was comparable to previous procedure**.** Mean DNA removal percentage following decellularisation with Pulmozyme + RNase was 99.9% (3.83 ng/mg). Mean DNA removal percentage with Benzonase was 99.8% (9.97 ng/mg). Histology staining showed complete decellularisation following either method. Benzonase was proven to be non-toxic to both cell lines used, and a one-way Anova test showed no significant difference in neither stress nor strain between acellular dermal matrix decellularised with either Benzonase or Pulmozyme + RNase T1. Benzonase was able to effectively decellularise dermis after prior removal of epidermis. It performed just as well as the combination of Pulmozyme + RNase T1, but represents significant advantages in terms of cost effectiveness, procurement and storage; Benzonase has been successfully used in the decellularisation of other tissues, thus would be better for Tissue Banking use. Switching to this combined DNase/RNase can have far-reaching consequences in the production of acellular human dermal matrix by NHSBT and in the treatment of patients requiring it.

## Introduction

In the UK there are approximately 3.8 million adult patients suffering from serious dermal wounds, costing the National Health Service (NHS) an estimated £8.3 billion a year (Guest et al. 2017). 1 million patients—equating to 2.0% of the adult population—suffer from ulcers of the lower limb; 326,000 patients suffer with diabetic foot ulcers, and 202,000 suffer with pressure ulcers (Guest et al. 2020). Around 30% of all wounds remain unhealed, costing the NHS £5.6 billion annually in wound management, healthcare assistants visits and district/community visits (Guest et al. 2017). Non-healing, full-thickness wounds, such as caused by diabetes or chronic ulcers, as well as those caused by burns or trauma, result in the destruction or removal of the whole of skin thickness. In order to prevent fibrosis or infection, skin grafting is often used, helping to re-establish skin continuity and provide protection from temperature, pathogens, and excessive water loss (Adams and Ramsey 2005; Braza and Fahrenkopf 2022). Skin grafts could either be full-thickness, consisting of the epidermis and entire dermis, or split-thickness, where only a portion of the dermis is retained. Whilst full-thickness grafts may be more metabolically active, their nutrient diffusion is slow and a more robust wound bed is required (Braza and Fahrenkopf 2022). Full-thickness grafts are therefore not recommended in poor wound beds such as chronic ulcers. Though advances in technology rendered synthetic scaffolds an option (Drury and Mooney 2003; Gilpin and Yang 2017), there are still obstacles in creating the biomechanical, biochemical, and structural complexities associated with a living tissue (Supp and Boyce 2005). Biological dermal scaffolds therefore offer the regenerative capabilities required in surgical procedures. Autografts of the patient’s own skin are preferred, and autologous new skin cell sheets may be cultured in the laboratory (referred to as Cultured Epithelial Autografts), but the process is costly, time-consuming, and requires long hospitalisation times for patients (Elseth et al. 2022) (Hadley et al. 2011). Furthermore, the need for a lengthy surgical procedure to remove an autologous graft before re-attachment, and the strain the NHS is currently under regarding bed availability (Bedford et al. 2020) make the use of allografts more practical. Non-viable (irradiated) skin allografts can be used as a mere biological dressing, but viable (cryopreserved) grafts which exhibit "graft take" are preferred as they gain a blood supply and are incorporated in the same way as an autograft (Hadley et al. 2011). However, immunogenicity is a concern and viable grafts are actively rejected due to the increased activity of the immune system’s dendritic Langerhans cells (Ang et al. 2014). Consequently, the removal of cellular components is required, not only to negate the immune response but also to allow host cells to repopulate.

Human decellularised dermal matrix (DCD) has been available commercially since 1995 (Shevchenko et al. 2010), and the process has been developed and validated in the UK by the National Health Service Blood and Transplant (NHSBT)’s Tissue Services as far back as 2013 (Hogg et al. 2013). Both epidermal and dermal cells are removed from donated skin originated from deceased tissue, preserving its dermal structure. DCD allografts have been reported to speed ulcer healing times (Cazzell et al. 2017), promote angiogenesis (Greaves et al. 2015), and reduce wound surface area by encouraging cell migration and proliferation (Greaves et al. 2013). They have also been reported to perform considerably better when compared with regular standard of care for chronic diabetic wounds (Armstrong et al. 2022). Furthermore, as DCD is acellular, it does not necessitate a Human Tissue Authority storage license in the UK (NHSBT 2022). Personal regenerative medicine is an emerging field in healthcare, allowing the development of personalised treatment tailored to the need of each patient (Arjmand et al. 2017). As DCD elicits no immune response, grafts can replace damaged tissue and become permanently populated by host cells, regenerating that tissue. There are several methods for decellularising tissue, including the use of organic solvents, detergents, or enzymes, with the latter proving more efficient and less harmful to tissue (Liu et al. 2019).

Effective digestion and removal of DNA from the donor is a key step in the preparation of a non-immunogenic dermis for transplant. At present, the enzymatic nuclease removal step of the decellularisation process validated by NHSBT is performed by incubating the tissue in a nuclease buffer consisting of RNase T1 and the recombinant human DNase Pulmozyme® (Genetech). The alternative to the combined use of Pulmozyme and RNase T1 is Benzonase® (Merck), a recombinant genetically engineered DNase/RNase originating from the Gram negative bacterium *Serratia marcescens* (Filimonova 2020), shown previously to be effective in dermis decellularisation (Bertasi et al. 2017; Fischer et al. 2018; Moore et al. 2015).

The use of the Pulmozyme + RNase T1 method involves calculating the exact amount of enzymes needed per surface area of tissue according to their specific activity, meaning up to 15 vials of RNase T1 may need to be mixed together under aseptic conditions before being added to the nuclease buffer. This is not only difficult to achieve under current Good Manufacturing Practice (cGMP) conditions in a clean-room environment, but is also very costly. Conversely, using the already more cost-effective option of Benzonase, which could be further aliquoted into smaller volumes, would not only be more cost-efficient but would also require a simpler single solution. Benzonase has been used by NHSBT’s Tissue and Eye Services (TES) in development studies to produce a range of decellularised tissues such as tendons, blood vessels, heart valves, amnion, and nerves. It has never been used to decellularise dermis, however, nor has its efficacy been directly compared with dermis decellularised with Pulmozyme + RNase T1. Previous pilot work within our collaborative network showed effective use of an alternative method with a single enzyme to digest DNA from the donor (Benzonase). There is therefore a need to keep abreast with new developments in the field of tissue decellularisation.

The aim of this study was to evaluate Benzonase’s ability to decellularise human dermis and to directly compare the DCD produced by this method against decellularised dermal matrix from the same donor(s), processed using Pulmozyme + RNase T1. As part of this validation study, acellular dermal matrix, produced by using both methods, was tested to assess possible effects on biochemical or biomechanical properties, and was contrasted with native, fully cellular skin.

## Methods

### Tissue

Skin used in this study was retrieved by NHS Blood and Transplant’s retrieval team from clinical donors, using a dermatome, and was chosen at random concerning sex and age of donor. It was processed according to the standard operating procedure protocol for cryopreserved skin of NHSBT’s Production team. The samples used were not fit for clinical use, but consent was given for research purposes.

### Decellularisation of skin samples

**Fig. 1:**
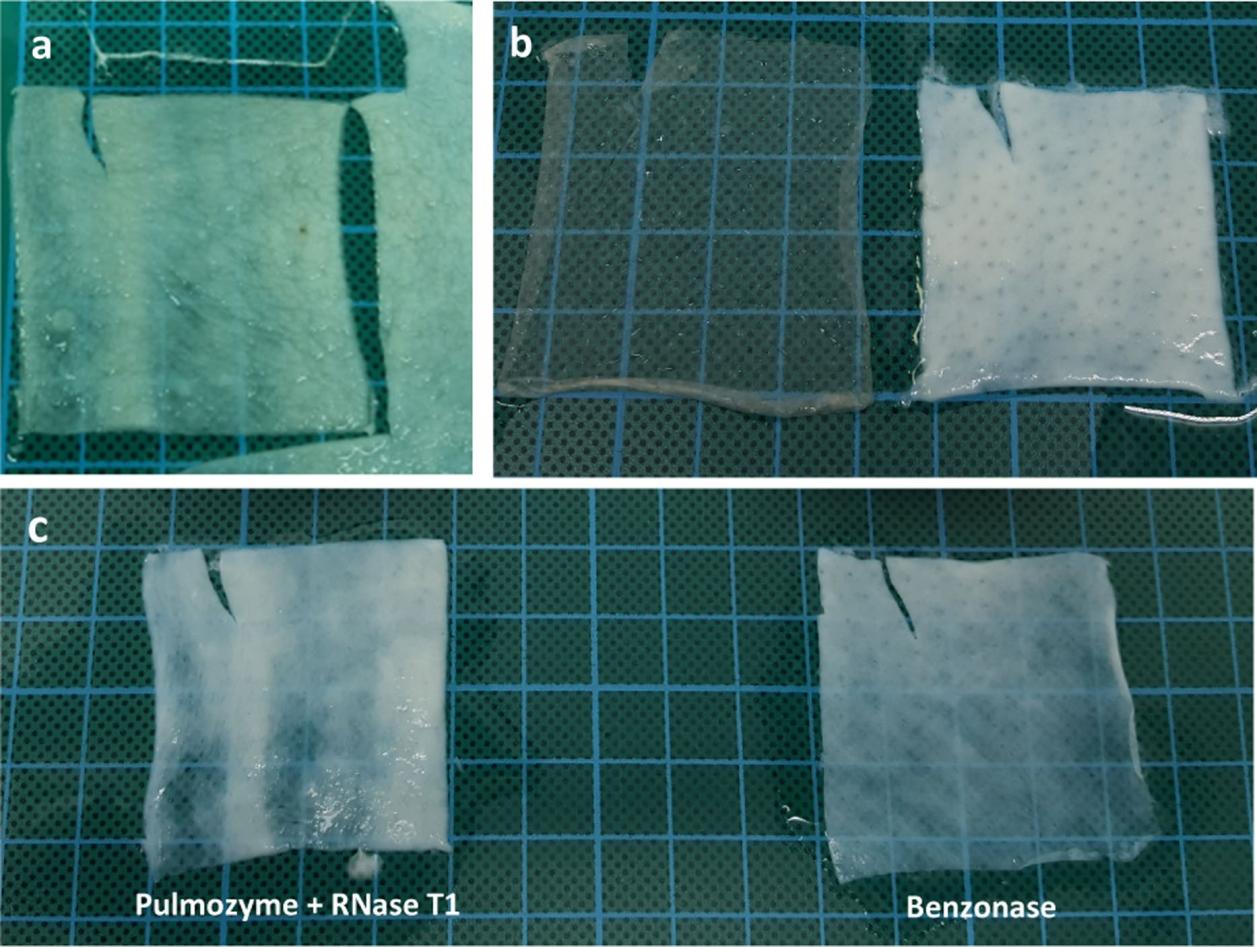
Stages in decellularisation of skin samples. **a** After glycerol was eluted off, skin was cut to 5 × 5 cm squares. **b** Following immersion in de-epideralising solution, epidermis layer (left) was removed. **c** Final products of dermis decellularisation

Skin samples were decellularised as described by Hogg et al. (Hogg et al. 2013). Skin batches used were previously decontaminated with an antibiotic solution when the skin was originally processed. As the samples were soaked in skin cryopreservation medium with 25% (w/w) glycerol (Serana) for storage in liquid nitrogen, following thawing, glycerol had to be eluted by 3 washes of 20 min in Dulbecco’s Phosphate Buffered Saline (PBS) (Sigma) with 10 KIU aprotinin (10,000 KIU/ml, Nordic Group) at room temperature (RT), 200 rpm on an orbital shaker (Fig. 1a). Squares measuring 5 cm x 5 cm were cut and the epidermis layer was removed by immersion in de-epideralising solution (Source BioScience) with 1% penicillin + streptomycin (10,000 U penicillin, 10 mg streptomycin per ml, Sigma), at 37 °C, 200 rpm on an orbital shaker, for 16–18 h [Fig. 1b]. Skin was then washed three times for 20 min with PBS + aprotinin, RT, 200 rpm, and cells were lysed using hypotonic buffer base (Source BioScience) with aprotinin and penicillin + streptomycin, at 4 °C, 150 rpm on an orbital shaker, for 16–24 h. Cellular organelle fragments were removed by incubating the skin samples in detergent buffer base (Source BioScience) with aprotinin and penicillin + streptomycin, RT, 200 rpm on an orbital shaker, for 24–26 h. Following the detergent stage, Skin was again washed three times for 20 min with PBS + aprotinin, RT, 200 rpm. DNA and RNA were further removed by using either separate DNase (Pulmozyme, Dornase alfa recombinant human deoxyribonuclease 1, 1000 U/ml, Roche) and RNase (T1, Easy-DNA, Invitrogen) or by using 100 U of a combination DNase/RNase (Benzonase, 27.1 U/μl, Novagen) in a nuclease buffer (Source BioScience) with aprotinin and penicillin + streptomycin, at 37 °C, 150 rpm on an orbital shaker, for 3 h (Fig. 1c). Skin was then washed three times for 20 min with wash buffer (Source BioScience) with aprotinin, RT, 200 rpm on an orbital shaker. Decellularised skin samples were kept in PBS with added aprotinin at 4 °C post decellularisation.

### DNA quantification

DNA levels in decellularise dermal matrix samples were measured using a commercially available kit (Easy DNA™ kit, Invitrogen) and compared to DNA levels in native skin. Small sections of skin were cut from cellular dermis, DCD decellularised with Pulmozyme + RNase T1, and DCD decellularised with Benzonase. Skin samples were dried in a 37 °C incubator for several days, then weighed and processed according to the manufacturer’s instructions. Extracted DNA levels were visualised with PicoGreen (Quant-iT PicoGreen dsDNA assay kit, Invitrogen) and measured on a fluorometer (FLX800 Microplate fluorescence assay reader, Biotek, UK). Samples’ total DNA concentrations were read with the aid of a NanoDrop™ 2000 Spectrophotometer (ThermoFisher Scientific), and a standard curve was produced in order to convert absorbance units to comparable DNA concentrations between DCD and cellular native skin from the same donor.

### Histology

Small biopsies of skin were removed from cellular native skin, from DCD decellularised with Pulmozyme + RNase T1, and from DCD decellularised with Benzonase. Biopsies were fixed in 4% buffered formaldehyde (VWR Chemicals) and placed in a plastic tissue embedding cassette. Skin and DCD biopsies were then washed in ethanol and xylene before being immersed in wax, as detailed in Table 1. Biopsies were embedded in a wax block using HistoStar wax embedder (Thermo Scientific), and were cut to produce 5 μm sections using a semi-motorised microtome (Histo-Core Multicut, Leica).

**Table 1:** Ethanol and xylene washes, prior to imbedding in wax, of skin and DCD biopsies for histology

Step	Solution	Time
1	4% buffered formaldehyde	2 h
2	Ethanol 70%	1 h
3	Ethanol 90%	1 ½ h
4	Ethanol 100%	1 h
5	Ethanol 100%	1 h
6	Ethanol 100%	1 h
7	Ethanol 100%	1 h
8	Xylene	1 h
9	Xylene	1 h
10	Xylene	30 min
11	Wax	2 h
12	Wax	2 h

Wax sections were mounted on slides and stained with either a fluorescent aqueous mounting media containing 4′,6-diamidino-2-phenylindole (DAPI), for fluorescent visualisation of DNA present in tissue, or with haemotoxylin and eosin, for visualising cell nuclei, extracellular matrix and cytoplasm. Slides were viewed and photographed on a Leica DMLA microscope system.

### In vitro* cytotoxicity*

Part 5 of ISO 10993 (Biological Evaluation of Medical Devices) (ISO. International Standard ISO 2009) describes test methods for the assessment of in vitro cytotoxicity of medical devices (2009). Contact cytotoxicity of both DCD samples decellularised with Pulmozyme + RNase T1, and DCD samples decellularised with Benzonase, was carried out. Small squares of DCD were attached into each well of a 24-well tissue culture plate with the aid of sterile Steristrip™ (3 M, UK). Steristrip™ with no attached tissue was also used as a negative control, whilst cyanoacrylate glue (RS Components, UK) was used as a positive control. DCD samples were then incubated with two cell lines—MG-63 (human osteosarcoma immortalised cells) and HSF (human skin fibroblasts)—for 48 h at 37 °C in a 5% CO_2_/air incubator. Once a confluent monolayer of cells was established, the wells were washed with 50% ethanol for 5 min, following by 70% ethanol for 5 min, the 100% ethanol for 5 min. 1 ml of filtered 20% Giemsa (VWR, UK) stain solution was added to each well for 5 min before rinsing with double-distilled water. After wells were air-dried, an inverted microscope (Leica 090-135-022, UK) was used to capture photomicrographs.

### Biomechanical (tensile) testing

Structural strength of cellular and DCD samples was assessed via the use of a materials testing machine (5ST, Tinius Olsen). Cellular and DCD samples were cut to an internationally recognised dumbbell shape by a Ray Run die cutter (DIN-5304 Type S3, Ray Ran, UK), to a length of 35 mm, and minimum and maximum width of 2–5 mm. Tissue samples were then clamped securely into the tensile testing machine and calibrated Vernier callipers (Digimatic CD-6″ C, Mitutoyo, UK) were used to measure thickness. The biomechanical properties of the tissues were assessed via the pull-to-break method, with a 100 N load cell. Test pulling speed was 100 mm/min and pre-load was set to 0.05 N. The Horizon computer assisted data acquisition and machine control system software, supplied with the Tinius Olsen tensile tester machine, was used to calculate the tissues’ ultimate stress and strain, with final data transferred and analysed on an Excel spreadsheet.

## Results

Skin samples from three donors were used. Samples were separated into three groups, and either decellularised through the methods described above (Pulmozyme with RNase T1, or Benzonase), or used as a cellular dermis control (Fig. 1) donor 1, n = 13; donor 2, n = 12; donor 3, n = 10 (Table 2). Within samples taken from each donor, no difference was found in the amount of DNA, therefore no donor-to-donor variation in DNA content can be reported (Table 3). Furthermore, a one-way Anova, run to compare the two nuclease protocols, found no significant difference between DNA traces in samples decellularised with either Pulmozyme RNase T1 or Benzonase (n = 25; *P* = 0.161; 95% CI = 1.48).

**Table 2:** Comparison of DNA in cellular dermis, acellular dermal matrix decellularised with Pulmozyme + RNase T1, and acellular dermal matrix decellularised with Benzonase

Sample	Donor	Biopsy weight (mg)	DNA/tissue (ng/mg)	% DNA remaining
Cellular dermis				
	1	4.93	4764.40	100
	2	8.99	9992.20	100
	3	8.74	3913.72	100
		*Mean*	*6223.44*	*100*
Acellular dermal matrix decellularised with Pulmozyme + RNase				
	1	6.58	0.64	0.013
	2	8.22	0.20	0.002
	3	8.74	10.64	0.271
		*Mean*	*3.83*	*0.095*
Acellular dermal matrix decellularised with Benzonase				
	1	7.09	7.31	0.153
	2	9.00	7.04	0.070
	3	7.84	15.56	0.397
		*Mean*	*9.97*	*0.20*

**Table 3:** Donor-to-donor variation in DNA found between cellular dermis, acellular dermal matrix decellularised with Pulmozyme RNase T1, and acellular dermal matrix decellularised with Benzonase

	Acellular dermal matrix decellularised with Pulmozyme + RNase	Acellular dermal matrix decellularised with Benzonase	Cellular dermis
n	13	12	10
Anova	*P* = 0.109	*P* = 0.281	*P* = 0.804
95% CI	0.923	0.759	0.609

### DNA quantification

Comparison of DNA present in control cellular dermis, DCD with Pulmozyme + RNase T1, and DCD with Benzonase is shown in Table 2. Cellular dermis was considered to have 100% of DNA remaining. The amount of DNA remaining in the DCD samples was expressed as a percentage of their corresponding control sample. Mean amount of DNA (ng/dry weight) remaining in tissues decellularised with Pulmozyme + RNase T1 was 3.83 ng/mg, whilst amount of DNA remaining in tissue after treatment with Benzonase was 9.97 ng/mg. Mean percentage of DNA remaining following decellularisation was 0.095% in samples treated with Pulmozyme + RNase T1, equivalent to 99.9% DNA removal. Mean percentage DNA remaining of samples treated with Benzonase was 0.2%, equivalent to 99.8% DNA removal.

**Fig. 2:**
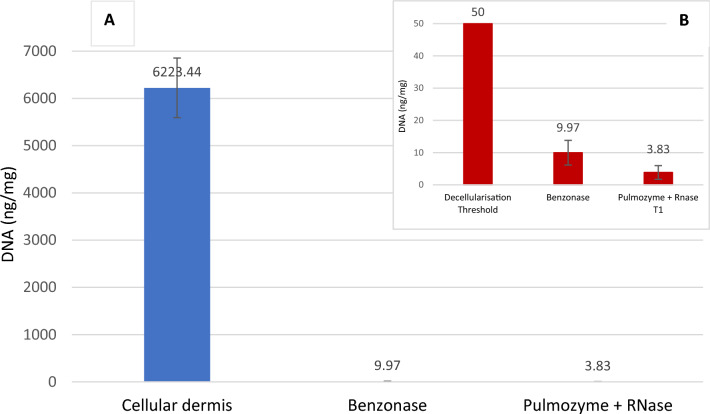
**A** Amount of DNA (ng/mg) per dry weight in cellular skin and decellularised dermal matrix (DCD). Mean amount of DNA in control cellular skin, DCD decellularised with Benzonase and DCD decellularised with Pulmozyme + RNase T1 from three donors; bars indicate standard deviation. **B** Mean amount of DNA in DCD decellularised with Benzonase and DCD decellularised with Pulmozyme + RNase T1, from three donors, compared to threshold amount set for decellularised tissue of 50 ng/mg DNA per dry weight. Bars indicate standard deviation

The amount of DNA in ng/mg per dry weight of tissue in control cellular dermis and decellularised dermis with either Pulmozyme + RNase T1 or with Benzonase is shown in Fig. 2.

### Histology

**Fig. 3:**
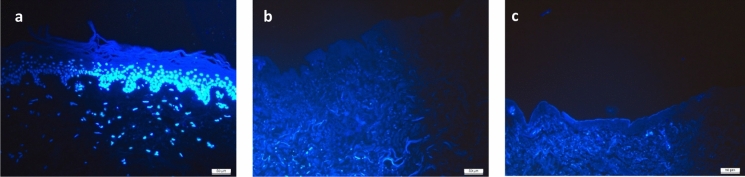
DAPI staining of histological sections. **a** Cellular skin, showing an upper layer of still-connected epidermis over intact dermis cells with nuclei, stained bright blue. **b** Decellularised dermal matrix with Pulmozyme + RNase T1, showing no intact cells or nuclei; (**c**) Decellularised dermal matrix with Benzonase, similarly, with no stained nuclei. Scale bars: 50 μm

4′,6-diamidino-2-phenylindole (DAPI) stain, for fluorescent visualisation of DNA present in the tissue, showed that in comparison with the control sample (Fig. 3a), DCD decellularised with either Pulmozyme + RNase T1 (Fig. 3b) or with Benzonase (Fig. 3c) showed no intact cells or stained nuclei, with only background autofluorescence present.

**Fig. 4:**
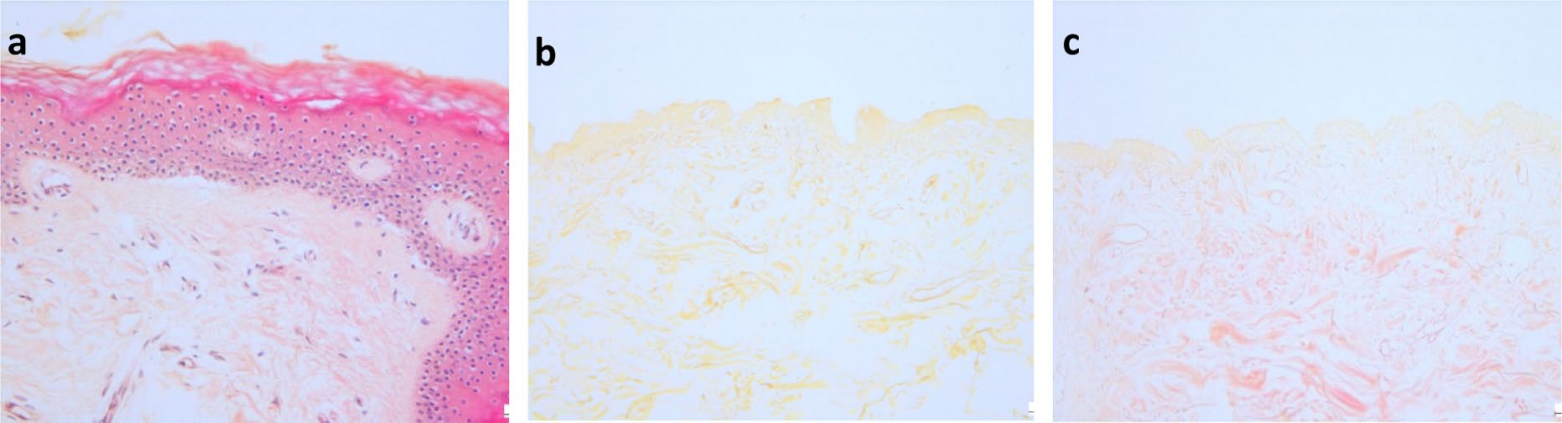
Haematoxylin and Eosin staining of histological sections. **a** Cellular skin, showing an upper layer of still-connected epidermis over intact dermis cells with nuclei. Cytoplasm and other tissue constituents, as well as extracellular material, are stained various shades of pink; cell nuclei are stained blue-purple. **b** Decellularised dermal matrix with Pulmozyme + RNase T1, showing no intact cells or nuclei; (**c**) Decellularised dermal matrix with Benzonase, similarly, showing no intact cells or nuclei. Scale bars: 50 μm

Similarly, staining with haematoxylin and eosin for the visualisation of cell nuclei and the extracellular matrix (Fig. 4a) showed that neither DCD samples treated with Pulmozyme + RNase T1 (Fig. 4b) nor those treated with Benzonase (Fig. 4c) displayed any discernible nuclei or intact cytoplasm.

### In vitro* cytotoxicity*

**Fig. 5:**
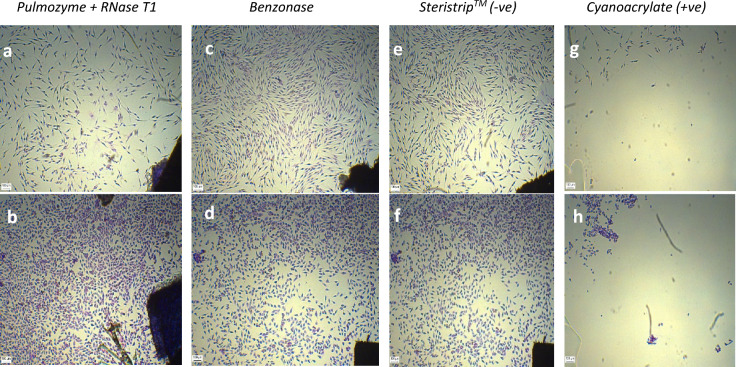
In vitro cytotoxicity. Decellularised dermal matrix (DCD) samples treated with Pulmozyme + RNase T1 are shown not to be cytotoxic to human skin fibroblasts (HSF) (**a**) or to human osteosarcoma immortalised cells (MG-63) (**b**). Similarly, HSF (**c**) and MG-63 (**d**) cells are shown to grow up to and contact DCD samples treated with Benzonase. Steristrip™ with no attached tissue was used as a negative control with HSF cells (**e**) and MG-63 cells (**f**). A clear inhibition zone is seen when HSF cells (**g**) and MG-63 cell (**h**) were grown in the presence of cyanoacrylate, used as a positive control. Scale bars: 100 μm

Cytotoxicity was used to assess possible cell death as a result of exposure to decellularised dermal matrix. Human osteosarcoma immortalised cells (MG-63) and human skin fibroblast cells (HSF) had shown no evidence of an inhibition zone when grown near DCD samples and no cell death was observed for either DCD samples treated with Pulmozyme + RNase T1 (Fig. 5a and b) nor those treated with Benzonase (Fig. 5c and d). Both DCD treatments resembled the Steristrip™ negative control (Fig. 5e and f) and not the positive cyanoacrylate control (Fig. 5g and h).

### Biomechanical (tensile) testing

Table 4 summarises the tensile stress analysis (expressed as ultimate force over surface area, in N/mm^2^) and strain analysis (expressed as deflection at break, in mm) that were carried out in a uniaxial tensile test on cellular skin and DCD samples. Mean stress for cellular skin was 2.014 N/mm^2^, 2.341 N/mm^2^ for samples decellularised with Pulmozyme + RNase T1, and 2.521 N/mm^2^ for samples treated with Benzonase. Mean strain for control cellular skin was 1.327 mm, 0.936 mm for samples treated with Pulmozyme + RNase T1, and 0.975 mm for samples decellularised with Benzonase alone. A one-way Anova showed there was no significant difference between cellular dermis, DCD samples treated with Pulmozyme + RNase T1, and DCD samples treated with Benzonase for neither stress nor strain.

**Table 4:** Ultimate strain and tensile stress in decellularised dermal matrix (DCD) and cellular skin

Sample		Stress	Strain
		Ultimate force/Area (N/mm^2^)	Deflection at break (mm)
*Cellular skin*			
	Mean	2.014	1.327
	SD	0.99	0.18
	95% CL	2.454	0.438
*DCD Pulmozyme* + *RNase T1*			
	Mean	2.341	0.936
	SD	0.65	0.18
	95% CL	1.035	0.291
*DCD Benzonase*			
	Mean	2.521	0.975
	SD	1.24	0.37
	95% CL	1.969	0.587
One-way Anova test		*P* = *0.802 Not significant P* > *0.05*	*P* = *0.181 Not significant P* > *0.05*

## Discussion

This study set out to compare the effectiveness of single-enzyme treatment with Benzonase for decellularising human dermis against the previously validated process of decellularisation using a combination of two expensive reagents RNase T1 and the DNase Pulmozyme. The acellular dermal matrix produced by using both methods was tested in order to assess the effects decellularising with Benzonase may have had on the tissues’ biochemical or biomechanical properties, and how it compared to tissues processed with Pulmozyme + RNase T1. In addition to comparing the decellularised dermal matrix produced by both methods to each other, the products were further compared to native, fully cellular skin for a complete validation study for tissue Banking and transplantation use. Benzonase was found to be just as effective at decellularising donated skin as Pulmozyme + RNase T1 when compared to native skin, and was shown to have no determinantal outcome in either tissue biomechanical properties or cell toxicity.

Angiogenesis, or the formation of new blood vessels in a transplanted tissue, has been proven to be of the utmost importance in wound healing (Greaves et al. 2015) (Veith et al. 2019), and is best achieved by the use of human tissue, containing both laminin and proteoglycans (Chien et al. 2021). However, in order to avoid inducing an immune response at the implant site, the tissue must be decellularised and all nucleic acids must be removed for the graft to take hold (Carlson et al. 2013) (Gilpin and Yang 2017). One of the most important criteria decellularised dermal matrix must fulfil in order to be considered acellular is that the extracellular matrix must have less than 50 ng of double-stranded DNA per mg of dry weight remaining at the end of the decellularisation process (Crapo et al. 2011), as it is unfeasible to remove all cellular components (Keane et al. 2015). The fluorometric dye PicoGreen was used to detect remaining DNA fragments in the tissues, as it has been shown to be far more sensitive than other standard spectrophotometric methods (Linthurst Jones et al. 2009). At the end of the decellularising process with either method, when compared to the fully cellular control skin, both tissues treated with Pulmozyme + RNase T1 or those treated with Benzonase only were successfully decellularised, as was reported before (Khan and Bayat 2019). Mean amount of DNA (ng/dry weight) remaining in tissues decellularised with Pulmozyme + RNase T1 was 3.83 ng/mg, whilst amount of DNA remaining in tissue after treatment with Benzonase was 9.97 ng/mg. This equalled 99.9% DNA removal by the Pulmozyme method and 99.8% DNA removal by Benzonase. Benzonase cleaves nucleic acids into short fragments of less than 5 nucleotides long which prevents them from amplifying (Amar et al. 2021), thereby it was able to perform as well as RNase, as was reported previously (Oristo et al. 2018). Benzonase was able to remove 99.8% of sample DNA, more than previously reported (Liu et al. 2019; Linthurst Jones et al. 2009; Amar et al. 2021), meaning remaining DNA was well below the 50 ng/mg requirement. This efficient DNA removal was equal to the reduction in DNA seen in samples treated with Pulmozyme + RNase T1.

Since the introduction of decellularised grafts, various methods had been suggested for the removal of genetic material whilst maintaining the structure of the extracellular matrix (ECM). However, no standardised method had been agreed upon (Bruyneel and Carr 2017). All cell removal techniques, whether physical, chemical or biological, are bound to affect the ECM, and care should therefore be taken to minimise the disruption. Ionic surfactants, such as sodium dodecyl sulfate (SDS), are widely used due to their efficiency in cell removal, however SDS has been noted to remove only around 90% of host DNA (Gilpin and Yang 2017) and is also cytotoxic over time. Skin, being a dense laminar tissue, could require long exposure time to such decellularisation agents if a detergent-only approach was to be taken (Costa et al. 2017). Nucleases, such as RNases and DNases, cleave nucleotide bonds and have been shown to aid in nucleotides’ elimination after cell lysis (Crapo et al. 2011) (McCrary et al. 2020). Decellularisation can consequently be improved by combining a chemical treatment with an enzymatic approach in order to restrict potential in vivo immunogenicity. It is well documented that nucleases are now often combined with detergents in order to expedite RNA and DNA removal from the ECM (Neishabouri et al. 2022) (Grauss et al. 2005) (Heath 2019) (Mendibil 2020), with Benzonase cited as being particularly effective due to its mid-sequence nucleotides-cleaving properties that aid with DNA fragmentation prior to removal (Petersen 2010).

As was mentioned above, the removal of cells and cellular organelles in order to prevent cellular components from initiating an immunological response upon transplantation is vital. In order to identify cellular components, used to determine the presence of cells in the biological scaffold, histological sections were stained with haematoxylin and eosin, and with DAPI, that binds to the AT regions of DNA. Histology analysis of samples treated with either decellularisation method showed a comprehensive removal of cell nuclei (Figs. 3 and 4). The tissues were rendered acellular by processing with either Pulmozyme and RNase or Benzonase, and no genetic material or cells were visible, corroborating the DNA analysis results. Except for the absence of cell nuclei, the histology images show no damage or change in the tissues’ structure, thereby maintaining the extracellular matrix’s native architectural characteristics (Figs. 3 and 4). As the three dimensional arrangement of the dermal tissue is crucial for aiding the process of wound healing (Khan and Bayat 2019), and as Benzonase was previously demonstrated to effectively decellularise dermal tissue without evidence of damage to the macro-architecture of the matrix (Khan and Bayat 2019; Greco et al. 2015), these results support the use of Benzonase as a decellularising agent.

Decellularised human dermal matrix can be used for various clinical procedures, such as aiding with the excision of cutaneous malignancies (Deneve et al. 2013), treatment of severe burns (Wainwright and Bury 2011), or scar contracture revision (Oh and Kim 2011). It is most often used to treat chronic leg ulcers, with complete healing achieved in 60% of patients (Greaves et al. 2013). With an estimated prevalence of between 1.5 and 3 per 1000 of chronic non-healing leg ulcers in the UK alone (Helliwell et al. 2017), and as decellularised dermal matrix allografts have been proven to reduce ulcer healing time by up to 50% (Cazzell et al. 2017; Reyzelman et al. 2009), it is little wonder NHSBT is required to supply surgeons with many decellularised allografts. As reagents used in the decellularising process may harm the biocompatibility or biomechanics of the extracellular matrix (Greco et al. 2015), and as residual substances from the process could impact the proliferation and adherence of cells (Reing et al. 2010), it is imperative that the decellularised allograft NHSBT supplies be not only acellular but non cytotoxic as well. Therefore, a standard cytotoxicity test, as stipulated by ISO 10993-5: Regulations of cytotoxicity in vitro (ISO. International Standard ISO 2009) and enforced in the UK by the Health Research Authority under EUTCD directive 2004/23/EC (Directives, E.U.T.a.C. Directive 2004), was carried out to test for possible cytotoxic effects. Along with possible allergic reactions in recipients even at small residual doses (Shaw et al. 2012), it has been previously reported that apoptosis can be induced in mammalian cells by overexposure to DNase (Fahmi, et al. 2020). However, there was no evidence of an inhibition zone when either human osteosarcoma immortalised cells or human skin fibroblast cells were grown near decellularised samples, and no cell death was observed for either DCD samples treated with Pulmozyme + RNase T1 nor those treated with Benzonase. Benzonase has been previously reported as non-cytotoxic to human skin fibroblasts, as well as human colorectal cancer culture HCT 116 (Filimonova 2020), baby hamster kidney cells, murine fibroblast cell line (Helliwell et al. 2017), human corneal epithelial line L-149, and the African Green Monkey Vero cell line (Liu et al. 2019). Furthermore, it has been deemed safe enough to be used in vaccine preparations (Fischer et al. 2018). Additionally, it has been shown to be undetectable in tissue following decellularisation (Liu et al. 2019).

In order for decellularised dermis to be fit for clinical use, it must be devoid of cells and free of genetic material, but still retain its original biomechanical properties. The decellularised human dermal matrix produced by NHSBT is retrieved as split thickness skin grafts, comprising of the epidermis and the upper part of the dermis, and includes the basement membrane and the structural proteins of the dermal matrix (NHSBT 2022). It is imperative that the extracellular matrix maintains its original biocompatibility and elasticity as wound healing relies on cellular proliferation and differentiation (Engler et al. 2006; Rehfeldt et al. 2007; Turner and Badylak 2015), as well as on stem cells recruitment (Brown et al. 2005; Wolf et al. 2012)—events which would only occur if the scaffold resembles cellular skin. The extracellular matrix consists of such molecules as fibronectin, vitronectin, laminin, glycosaminoglycans and collagen IV, in a three-dimensional structure (Agrawal et al. 2011). The main collagen of the extracellular matrix—collagen IV—is found in the basement membrane, and due to its assembled protomers superstructure is flexible and can bend (Abreu-Velez and Howard 2012). It has been suggested that high levels of metalloproteinase, found in chronic wound matrices, break the collagen in the applied scaffold allograft and release bound growth factors, which in turn increase angiogenesis and cell growth, and reduce inflammation (Turner and Badylak 2015). Collagen is also the skin’s primary mechanostructural element, conferring not only proteolytic defence but also tensile strength to the tissue (Greco et al. 2015). In this study, uniaxial tensile testing of both the cellular and decellularised tissues was chosen in order to compare potential structural damage following the decellularisation process. Tissue samples decellularised by either Pulmozyme + RNase T1 or by Benzonase were tested for stress and strain, and were compared to whole, cellular skin samples. The data indicated that the neither decellularisation process had any effect on the tissues’ tensile strength, and a one-way Anova showed no significant difference between cellular dermis, DCD samples treated with Pulmozyme + RNase T1, or DCD samples treated with Benzonase for neither stress nor strain. These data correspond to previously reported findings by Hogg et al. which demonstrated that the collagen IV-containing basement membrane remained intact after the decellularisation process (Hogg et al. 2015) and that decellularised dermal matrix had shown the same biomechanical properties as cellular skin (Hogg et al. 2013). This supports preceding evidence that the decellularisation process does not cause damage to the extracellular matrix’s macro-architecture in porcine dermis (Greco et al. 2015) and in airway epithelial grafts (Hamilton, et al. 2020), and that no biomechanical difference were found between cellular dermis and decellularised dermal matrix when samples were treated with Benzonase (Greaves et al. 2015; Helliwell et al. 2017).

Whilst our data show that Benzonase is as efficient in digesting DNA and RNA as Pulmozyme + RNase T1 during the decellularisation of human skin, decellularising with Benzonase is much more cost-effective (Wen et al. 2016; Yuan et al. 2019). Pulmozyme (dornase alfa) is a recombinant human deoxyribonuclease, known to improve sputum surface properties, thereby easing the clearance of sputum (Scala et al. 2017). It is particularly used in the treatment of patients suffering from cystic fibrosis (Jones and Wallis 2010). As such it is classed as medication, requiring prescription in order to obtain, and is far more expensive than Benzonase, as was noted before (Vafaee et al. 2018). Furthermore, in keeping with our results, it has been demonstrated that whilst Benzonase has similar decellularising efficacy, the amount needed could be several time less than Pulmozyme (Filimonova 2020). Additionally, even if a more costly—research-grade—Benzonase is used, the possibility of aliquoting and storing it for further use reduces its initial cost tremendously. As a not-for-profit organisation, NHSBT could benefit from lowering the cost of decellularised skin production, thereby keeping the price of allografts supplied to hospitals low. Benzonase was reported to show broad activity towards DNA substrates (Amar et al. 2021) and efficiently degrade both RNA and DNA in a wide range of pH and temperatures (Wen et al. 2016; Ye et al. 2011). Moreover, as only a small amount of Benzonase is needed for efficient DNA and RNA digestion (Liu et al. 2019), a decellularisation process using Benzonase is less vulnerable to operator calculation errors in comparison to calculations needed for assessing the correct amount needed for the use of RNase T1 in each batch.

There were a number of limitations to this study, chiefly the low number of replicate skin samples. However, as this was a pilot study to determine further action that might be taken considering the use and validation of Benzonase as a decellularisation agent, a small number of replicants is acceptable. There was also some degree of variation within and between samples in terms of DNA content and strength testing. These were primarily due to the use of donated human tissues which are, by nature, heterogeneous both between patients and within the same individual, depending on which part of the body the donation was taken from. Future analysis of dermal collagen could utilise Masson’s trichrome staining (Hong et al. 2020) to further quantify comparisons between DCD samples and cellular skin, or mass spectrometry to test for traces of endonucleases in skin (Munem et al. 2021) or to further evaluate the effect on extracellular matrix proteins (Randles et al. 2017). Future research could also be directed towards scaling up the decellularisation process with Benzonase, as described here, in order to tailor it to full production with NHSBT’s Tissues and Eye Services Production department. Further research would be needed to ascertain that aliquoting Benzonase does not lower its efficacy, either through storage conditions or by interaction with the polypropylene in the Eppendorf tubes. Furthermore, The process would have to be validated by the Human Tissue Authority via a Preparation Process Dossier (Authority 2022) to establish that it does not render tissue clinically ineffective or harmful.

## Conclusion

This study has shown that Benzonase was able to remove 99.8% of DNA from decellularised human skin, well below the 50 ng/mg required, and was just as effective as Pulmozyme + RNase T1. Furthermore, Benzonase decellularised dermal tissue without any evidence of damage to the macro-architecture of the extracellular matrix, nor was there evidence of cytotoxicity when acellular dermal matrix decellularised with Benzonase was introduced to either human skin fibroblast cells or human osteosarcoma immortalised cells. Uniaxial tensile testing, assessing potential structural damage following the decellularisation process, found that processing with Benzonase had no effect on the tissue’s tensile strength for neither stress nor strain, akin to both dermal matrix decellularised with Pulmozyme + RNase T1 and to fully cellular skin. As Pulmozyme is sold as a medicinal product for the treatment of cystic fibrosis, it requires a medical prescription for procurement; it is also expensive, especially with the added cost of RNase T1. Benzonase can perform just as well, and if aliquoted, at a fraction of the price. It has also been shown to be more ‘operator-friendly’, with a wide working range of pH and temperatures. Whilst Benzonase has been used to decellularise other tissues in the past, it has not been used in the production of decellularised skin. Demonstrating this endonuclease’s ability to effectively remove both DNA and RNA from human skin, whilst maintaining the tissue’s structural and biochemical integrity, can have far-reaching consequences for the production of DCD by NHSBT’s TES, and for treatment of various conditions requiring skin allografts.
